# Prognostic Factors in Neuroendocrine Neoplasms of the Rectum

**DOI:** 10.3390/cancers17172841

**Published:** 2025-08-29

**Authors:** Frederike Butz, Charlotte Friederike Müller-Debus, Flora Georgina Ecseri, Gianna Sophia Mani, Elif Akgündüz, Agata Dukaczewska, Peter Richard Steinhagen, Uli Fehrenbach, Catarina A. Kunze, Henning Jann, Johann Pratschke, Eva Maria Dobrindt, Martina T. Mogl

**Affiliations:** 1 Department of Surgery, Campus Charité Mitte | Campus Virchow Klinikum, Charité—Universitätsmedizin Berlin, Corporate Member of Freie Universität Berlin and Humboldt-Universität zu Berlin, 10117 Berlin, Germany; 2 Department of Hepatology and Gastroenterology, Campus Charité Mitte | Campus Virchow-Klinikum, Charité—Universitätsmedizin Berlin, Corporate Member of Freie Universität Berlin and Humboldt-Universität zu Berlin, 10117 Berlin, Germany; 3 Department of Radiology, Charité—Universitätsmedizin Berlin, Corporate Member of Freie Universität Berlin and Humboldt-Universität zu Berlin, 10117 Berlin, Germany; 4 Department of Pathology, Charité—Universitätsmedizin Berlin, Corporate Member of Freie Universität Berlin and Humboldt-Universität zu Berlin, 10117 Berlin, Germany; 5 Department of Internal Medicine, DRK Kliniken Berlin Köpenick, 10117 Berlin, Germany

**Keywords:** rectal neuroendocrine tumors, neuroendocrine tumors of the rectum, prognostic factors, oncological outcome

## Abstract

Background/Objectives: Neuroendocrine neoplasms (NENs) of the rectum (rNEN) are a rare and heterogeneous group of tumors that can vary greatly in their biological behavior, from benign to highly aggressive. While small and well-differentiated tumors can often be handled endoscopically and still face a very good prognosis, tumor size, and grade, as well as lymph node and distant metastasis, are known risk factors for impaired prognosis. This study aimed at the identification of further prognostic factors in rNEN. Methods: A retrospective analysis of patients treated for rNEN at the ENTES Center of Excellence at Charité—Universitätsmedizin, including n = 121 patients, was performed to identify risk factors for recurrence, progression, and impaired outcome. Results: Progression-free survival (PFS) and overall survival (OS) differed significantly according to tumor grade (*p* < 0.001). In rNET patients undergoing surgery, the Ki-67 index and distant metastases were independent risk factors for shorter PFS. Among stage I rNETs, 10 patients developed disease recurrence, associated with lymphatic invasion (1.9% vs. 30.0%, *p* = 0.008) and higher Ki-67 values (2 (1–6) vs. 2 (1–16), *p* = 0.054). Conclusions: Tumor grade and presence of metastases represent important predictive factors in rNEN. Notably, even small, early-stage rNETs can harbor a risk of recurrence when unfavorable pathological features are present, highlighting the importance of tailored, risk-adapted surveillance strategies to optimize patient outcomes.

## Simple Summary

Neuroendocrine neoplasms of the rectum are rare tumors that vary from harmless to very aggressive. While small tumors are often treated successfully with simple endoscopic procedures, more aggressive therapy is needed for advanced tumors and when high-risk features are present. This study looked at 121 patients to identify factors linked to disease recurrence and survival. It showed that tumors with a higher grade or distant metastases were more likely to recur or progress. Notably, even small, early-stage tumors showed local or distant recurrence when certain risk factors—such as lymphatic vessel invasion or a high proliferation index—were present. These findings highlight the importance of personalized treatment strategies based on individual tumor characteristics to achieve the best possible outcomes.

## 1. Introduction

The incidence of neuroendocrine neoplasms (NENs) of the rectum (rNEN) has been increasing in recent decades, and one possible explanation is the widespread implementation of colorectal cancer screening via colonoscopy [1,2]. rNENs comprise a heterogeneous group of rare tumors with great variation in their biological behavior: from mostly indolent to highly aggressive forms. They are classified according to the WHO classification into well-differentiated neuroendocrine tumors (NET) (either G1, G2, or G3 grading based on Ki-67 index and mitotic count), poorly differentiated and highly aggressive neuroendocrine carcinoma (NEC), and mixed neuroendocrine–non-neuroendocrine neoplasm (MiNEN) [3]. Furthermore, tumor staging for well-differentiated rNETs is generally based on the TNM system established by the American Joint Committee on Cancer (AJCC) [4].

While well-differentiated rNETs typically carry a favorable prognosis with low risk of recurrence or development of metastases, poorly differentiated NECs are highly aggressive and associated with poor survival, with reported 5-year survival rates as low as 10% [5]. Tumor size, differentiation, grading, and stage have been shown to correlate with the risk of lymph node involvement, distant metastases, and survival [5,6,7,8]. As such, current treatment recommendations are based on these parameters [8]. In localized rNETs smaller than 1 cm, the risk of lymph node or distant metastases is considered low, and endoscopic treatment is usually regarded as sufficient [8]. Conversely, oncological resection is recommended in tumors larger than 2 cm, with higher grade (G2 or G3), and those presenting lymphatic or microvascular invasion [8]. However, recent evidence suggests that even small, early-stage rNETs may carry a risk of developing lymph node metastases or disease recurrence, challenging the assumption that these lesions invariably follow a benign course [9,10]. In addition, a key factor for sufficient endoscopic treatment for small rNETs is the proper identification of the lesion to choose an appropriate resection technique and achieve complete pathological resection. Unfortunately, misidentification and misclassification of rNETs during colonoscopy are common and occur in 41% up to 82% [11,12]. Therefore, incomplete resection rates remain elevated [11], while their prognostic impact in small rNETs are still discussed [13,14]. Identification of risk factors for adverse oncological outcomes and the development of recurrence and metastases in small rNETs is crucial to identify the individuals requiring additional treatment and to guide optimal, individualized management.

Despite increasing recognition of rectal NETs, data on rectal NECs and MiNENs remain especially limited. These entities are rare, often grouped together with NETs in large registry analyses, and frequently misclassified due to diagnostic challenges and sampling bias [15,16].

In this study, we present a single-center outcome analysis of patients with rNENs, including NETs, NECs, and MiNENs. We aimed to identify risk factors associated with disease recurrence in rNENs, with a particular focus on small, localized rNETs. We hypothesized that even among small (≤10 mm), localized rNETs, adverse pathological features such as lymphatic invasion and elevated Ki-67 index were associated with disease recurrence. We further hypothesized that in the broader rNEN cohort, pathological characteristics such as tumor grade and pathological subtype remained predictors of progression-free and overall survival.

## 2. Materials and Methods

### 2.1. Patient Selection and Data Collection

Patients with rNEN who were treated at the European Neuroendocrine Tumor Society (ENETS) Center of Excellence between January 2009 and June 2022 were identified from the Charté Comprehensive Cancer Center database. Exclusion criteria were primary tumor locations other than the rectum and cases without follow-up data (n = 10).

Demographic and clinical data were retrieved from clinical charts, and pathological reports were reviewed for pathological data, including Ki-67 index, grading, tumor differentiation, tumor size, pathological tumor (T) stage, pathological nodal (N) stage, resection margin status (R status), lymphatic (L), microvascular (V), and perineural (Pn) invasion. However, information about Pn invasion was missing or could not be determined in most patients, and the parameter was therefore excluded from the analyses. As the pathological N stage could only be determined for patients undergoing lymphadenectomy, it was stated as unknown Nx in patients undergoing localized resection. The Ki-67 index was determined during routine pathological examination according to the in-house protocol. In general, immunohistological staining for Ki-67 was performed using a mouse monoclonal antibody (clone MIB-1; 1:50, Dako/Agilent, Santa Clara, CA, USA) on the Ventana Benchmark Ultra plus autostainer instrument (Ventana Medical Systems, Inc., Tucson, AZ, USA) according to the manufacturer’s protocol. 3, 3′-diaminobenzidine peroxide substrate (DAB +) from the “ultraView Universal DAB detection kit” (Ventana, Tucson, AZ, USA) was used as a chromogen. Quantification of Ki-67-positive tumor cell nuclei was performed relative to the total number of tumor cell nuclei. Assessment was carried out in hotspot areas, which were selected at low microscopic magnification. Positive tumor cells were counted in at least one high-power field, comprising a minimum of 500 tumor cells, and were performed either manually or using computer-assisted image analysis. All examiners were board-certified pathologists.

Preoperative tumor stage was defined according to the 8th edition of the American Joint Committee on Cancer (AJCC) [4] and tumor grading according to the WHO grading system [3]. Endoscopic resection types included simple polypectomy, endoscopic mucosal resection, endoscopic submucosal dissection, and endoscopic full thickness resection. Surgical resection included transanal minimally invasive surgery and oncological low anterior resection with total mesorectal excision, as well as abdominoperineal resection. Non-surgical treatments included chemotherapy, peptide receptor radionuclide therapy (PRRT), and best supportive care (BSC). Disease recurrence was determined after clinical and radiological assessments based on Response Evaluation Criteria in Solid Tumors (RECIST version 1.1) [17].

### 2.2. Statistical Analysis

Continuous variables are expressed as medians with ranges, while categorical variables are reported as absolute frequencies and percentages. Comparisons of continuous variables were conducted using the Mann–Whtiney U test, and Fisher’s exact test was applied for comparisons of categorical variables. Progression-free survival (PFS) and overall survival (OS) were estimated using the Kaplan–Meier method, and differences were compared using the log-rank test. PFS was defined as the time from first treatment to the first documented postoperative disease progression, and OS as the interval from first treatment to death. Patients not reaching the respective endpoint or lost to follow-up were censored at the date of last follow-up. Subgroup analysis of NEN according to histological type (NET, NEC, and MiNEN) was performed to distinguish between these biologically different tumor types. Univariate Cox regression analysis was performed for the identification of factors associated with PFS and OS in rNET patients undergoing primary resection. Results are displayed as hazard ratio (HR) with 95% confidence interval (95% CI). For multivariable regression analysis identifying risk factors for impaired PFS, factors with significant effect in univariate analysis showing a *p*-value < 0.001 were included. As only n = 18 events (disease progression) were recorded, the number of variables included in the multivariable analysis was limited to 2. As only n = 5 deaths occurred in the rNET cohort who underwent primary resection, multivariable analysis was not performed.

A *p*-value below 0.05 was considered statistically significant. Missing data were specified and excluded from analysis by analysis. To address missing data regarding lymphatic and microvascular invasion, we applied multiple imputation using the Fully Conditional Specification (FCS) method with 5 imputations, including the variables T and N stage in the imputation model. Logistic regression was performed to analyze the association of lymphatic and microvascular invasion and disease recurrence (see Supplementary Table S1). The imputed datasets were analyzed separately, and the results were pooled according to Rubin’s rules. To assess the robustness of our findings, we additionally conducted complete-case analyses and compared them with the pooled estimates from the imputed datasets (see Supplementary Table S2). Results from multiple imputation were consistent with those obtained from complete-case analyses. Therefore, results from complete-case analyses were included in the main results section.

All statistical analyses were conducted using SPSS Statistics, version 29 (IBM Corp., Armonk, NY, USA).

The study received approval from the local institutional ethics committee (EA2/064/09) and was conducted in accordance with the principles of the Declaration of Helsinki. Informed consent was waived due to the retrospective and non-interventional nature of the study.

## 3. Results

### 3.1. Patient Characteristics

The study included a total of 121 patients with rNEN, and characteristics are shown in Table 1 for all rNEN patients and specified by pathological type. Regarding the pathological type, n = 84 patients (69.4%) were well-differentiated NETs, n = 26 patients (21.5%) had poorly differentiated NECs, and n = 11 had MiNEN. Accordingly, 57 patients (47.1%) had a G1 tumor, 30 patients (24.8%) a G2, and 34 patients (28.1%) a G3 tumor throughout the whole study cohort. Most patients had localized stage I tumors (52.1%), while 26.4% already had distant metastases at diagnosis. The proportion of localized tumors was remarkably higher in NET patients (n = 62, 73.8%) than in NEC (n = 0) and in MiNEN patients (n = 1, 9.1%). Nearly 80% of the whole cohort (n = 96) received either endoscopic or surgical resection of the primary. Of these, 63.9% (n = 62) achieved complete resection, and n = 24 patients (26.8%) underwent a second resection. Moreover, 16.5% (n = 20) received primary chemotherapy, 3.3% PRRT (n = 4), and for n = 1 patient (0.8%) (a NEC patient), best supportive care was chosen primarily.

**Figure 1:**
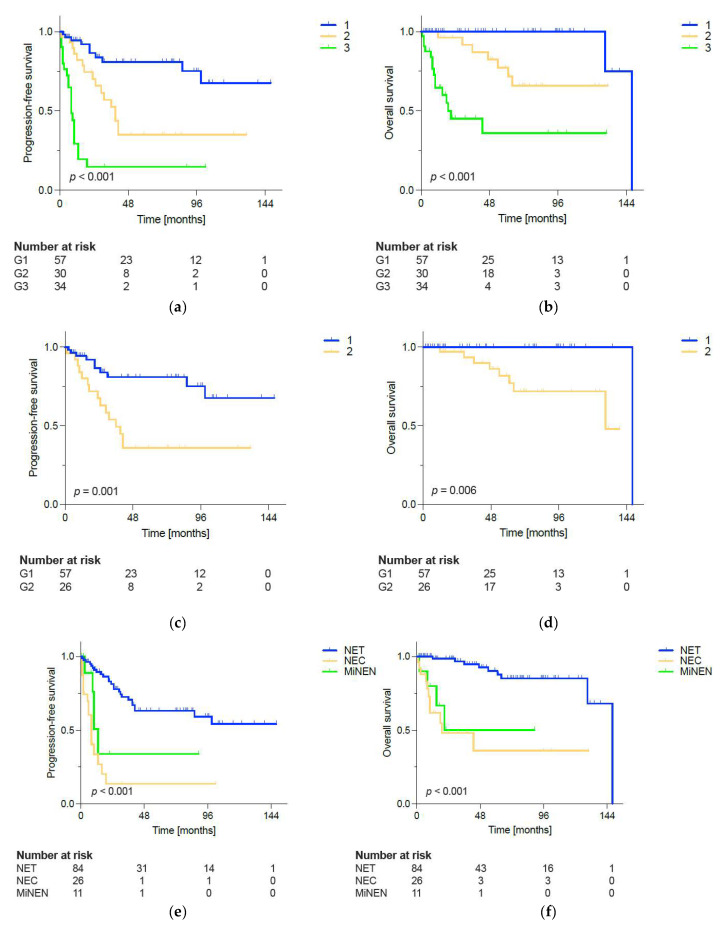
Progression-free survival (PFS) (**a**,**c**,**e**) and overall survival (OS) (**b**,**d**,**f**) in patients with rNENS according to tumor grading (**a**–**d**) and pathological type (**e**,**f**). (**a**) PFS differed significantly by grading in all rNENs with 5-year PFS of 80.9% (G1), 35.1% (G2), and 14.7% (G3), *p* < 0.001. (**b**) OS also varied by grading, with 5-year OS of 100% (G1), 77.4% (G2), and 36.0% (G3), *p* < 0.001. (**c**) Among rNETs, PFS was shorter in G2 compared to G1 tumors (5-year PFS 80.9% in G1 vs. 35.9% in G2, *p* = 0.001). (**d**) OS likewise differed between G1 and G2 rNETs (5-year OS 100% (G1) vs. 81.8% (G2), *p* = 0.006). (**e**) PFS varied by pathological subtype with 5-year PFS of 63.1% in rNETs, 13.4% in rNECs and 33.9% in rMiNEN, *p* < 0.001. (**f**) OS also differed according to pathological subtype, with 5-year OS of 90.3% in rNETs, 36.0% in rNECs, and 50.0% in rMiNENs, *p* < 0.001. Survival rates were compared using the log-rank test. Ticks mark censored data.

Throughout the whole study cohort, median estimated PFS was 94, and median estimated OS was 148 months. As shown in Figure 1, grading was significantly associated with PFS and OS and gradually decreased with increasing grade throughout all pathological subtypes: 5-year PFS was 80.9% in G1, 35.1% in G2, and 14.7% in G3 patients (*p* < 0.001). In addition, 5-year OS in G1 was 100%, 77.4% in G2, and 36.0% in G3 patients (*p* < 0.001). When only focusing on rNETs, PFS and OS differed significantly between different tumor grades, too, with longer PFS and OS in G1 rNETs than in G2 rNETs (compare Figure 1c,d). PFS and OS were also associated with the pathological subtype of rNENs: rNETs had significantly longer PFS and OS than rNECs and rMiNENs (5-year PFS: 63.1% (rNETs) vs. 13.4% (rNEC) vs. 33.9% (rMiNEN), *p* < 0.001 and 5-year OS: 90.3% (rNET) vs. 36.0% (rNEC) vs. 50.0% (rMiNEN), *p* < 0.001) (see Figure 1e,f).

### 3.2. rNEN Patients Undergoing Primary Resection

Characteristics of all rNEN patients who underwent primary resection and according to histological type are shown in Table 2. N = 16 patients (16.7%) had poorly differentiated NECs, and n = 5 patients (5.2%) showed a MiNEN. While n = 63 patients (65.6%) of the whole cohort had stage I disease, 9 patients (9.4%) had stage 2, 13 patients (13.5%) had stage 3, and 11 patients (11.5%) had stage IV disease. The proportion of stage I tumors in rNETs (n = 62, 82.7%) was higher than in rNECs (n = 0) and rMiNENs (n = 1, 20.0%). Most rNET patients had G1 tumors (n = 55, 73.3%), while n = 3 (60.0%) of all rMiNENs were G2 and n = 2 (40.0%) were G3 tumors. In addition, the majority of rNET patients were treated endoscopically (n = 63, 84.0%), while rNEC and rMiNEN patients mostly had surgical primary resection. R1 resection occurred in n = 25 patients (26.0%), and unclear resection margins (Rx) were stated in n = 9 patients (9.4%). A second resection was performed in about one quarter of the total cohort (n = 24, 25.3%); in n = 18 cases (75.0%) it was endoscopic, while surgery was chosen in n = 6 (25.0%) cases. Lymph node metastases were found in most rNECs (n = 11, 68.8%), while the pathological N stage was stated as unknown in the majority of rNET patients (n = 63, 84.0%) who underwent endoscopic resection, as no lymphadenectomy was performed and neither was indicated based on clinical staging. Observing the whole rNEN cohort, local recurrence occurred in n = 11 (11.5%) patients, while n = 26 (27.1%) patients developed lymph node or distant metastases during follow-up. Remarkably, 81.3% (n = 13) of rNEC patients had distant disease recurrence or progression.

In order to identify risk factors for worse PFS and OS in patients eligible for primary resection focusing only on rNETs, Cox regression analysis was performed (see Table 3 and Table 4). Increasing Ki-67 index and therefore tumor grading, as well as increasing tumor size and positive distant metastases, were associated with shorter PFS in univariate analysis. Multivariable analysis identified increasing Ki-67 index (HR 1.071 (1.017–1.128), *p* = 0.009) and distant metastasis (5.032 (1.615–15.683), *p* = 0.005) as independent risk factors for shorter PFS. Regarding OS, increasing Ki-67 index was associated with impaired OS in rNET patients undergoing primary resection. However, the low number of events (n = 5) weakens the statistical quality, and multivariable analysis was waived.

### 3.3. Disease Recurrence in Stage I rNETs

**Figure 2:**
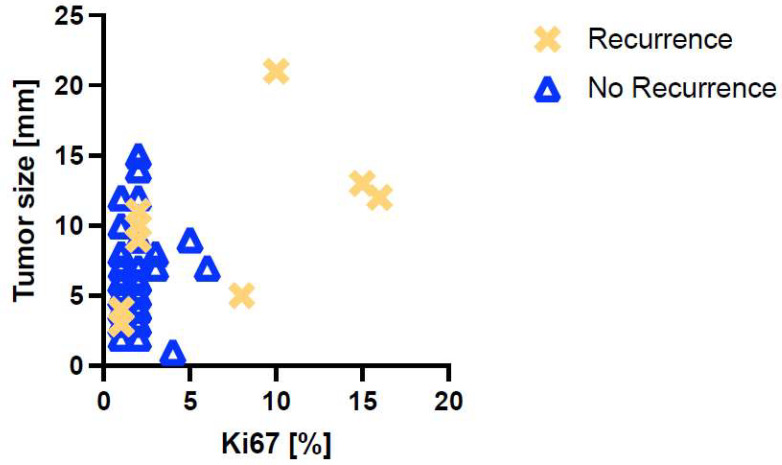
Small rNETs with disease recurrence tend to be larger and have a higher Ki-67 index. The scatterplot shows the distribution of Ki-67 (%) and tumor size (mm) in small rNET patients, stratified by disease recurrence status. Each data point represents an individual case. Patients with recurrence are indicated by yellow crosses and patients without recurrence by blue triangles.

Focusing on stage I rNETs, the oncologic outcome was favorable with a 5-year PFS of 86% and a 5-year OS of 100%. To further identify risk factors for disease recurrence in these small, localized rNETs, a comparison between patients with and without disease recurrence was performed (see Table 5). In total, 10 patients developed either local and/or distant disease recurrence during the median follow-up of 45 (2–145) months. In addition, n = 1 NET-related death occurred among rNET patients with recurrence. When comparing clinicopathological characteristics between rNET patients with and without recurrence, lymphatic invasion occurred more often in patients with recurrence (1.9% vs. 30.0%, *p* = 0.008), while other characteristics showed no significant differences. Yet, as graphed in Figure 2, rNET patients with recurrence tended to have higher Ki-67 values (2 (1–6) vs. 2 (1–16), *p* = 0.054), and the proportion of G2 tumors was higher (13.5% vs. 40.0%, *p* = 0.067), although statistical significance was not met.

When looking into the patients who showed local recurrence, all recurrent rNETs could be treated endoscopically, achieving an R0 resection. Interestingly, of the five patients with distant disease recurrence, n = 4 had a T1b tumor larger than 10 mm and n = 4 a G2 tumor. However, no lymphatic invasion was found in patients with distant recurrence.

## 4. Discussion

The current study presents outcome data from a large single-center cohort of rNEN, confirming the association of tumor grading with PFS and OS in rectal NET, NEC, and MiNEN. In rNET patients undergoing primary resection, increasing Ki-67 index and distant metastases were confirmed as independent risk factors for shorter PFS. Localized rNETs smaller than 2 cm were at low risk for local recurrence or development of lymph node or distant metastases. Lymphatic invasion, tumor size larger than 10 mm, and G2 tumors were associated with disease recurrence. Nonetheless, even in the event of local or distant recurrence, the overall outcome remained favorable.

In line with previous literature, most patients in the study cohort had small tumors (<2 cm) without evidence of lymph node or distant metastases. However, while in the presented study cohort, the proportion of stage I tumors was around 52%, mainly larger amounts have been reported previously [7,18,19]. For example, in their work, Zeng et al. describe the proportion of stage I tumors as high as 82% [18]. This difference may be due to a selection bias, as the current study did not include registry data; all patients were treated for rNEN at an ENETS Center of Excellence. Therefore, an unknown amount of small, endoscopically diagnosed, and completely resected rNETs might not have been referred for follow-up or treatment.

In the here-presented study cohort of small rNETs, 10 out of 62 patients had recurrent disease, with n = 7 patients showing local recurrence—a significantly higher recurrence rate than previously reported. For example, Kang et al. identified in their meta-analysis from 2019 a recurrence rate of only 0.3% in small rNETs [20]. Similar findings were reported by Dabkwoski et al. who summarized recurrence rates from 0.015 to 0.035 depending on endoscopic resection techniques in small rNETs [21]. Hence, as already stated above, some kind of confounding bias is most likely leading to a possible reduced inclusion of “low-risk” rNET patients.

In the whole study cohort, tumor grading was associated with both shorter PFS and shorter OS. In addition, in patients undergoing primary tumor resection, especially G3, it was an independent risk factor for shorter PFS. Grading based on the Ki-67 index is an established prognostic factor for digestive NETs [22]. In their study, Capurso et al. evaluated the prognostic role of the WHO grading system and confirmed its ability to predict PFS and OS in a cohort of 100 rNENs [5]. Furthermore, they were able to show that tumor stage was predictive for oncologic outcome, too. The here-presented data showed an association of distant metastases corresponding to stage IV disease with impaired PFS. Therefore, accurate tumor staging and grading according to existing guidelines is of utmost importance to select adequate treatment and follow-up regimens [8].

rNECs are characterized by their poor differentiation, high proliferative activity, and aggressive clinical behavior. Consistently, the prognosis for these tumors remains extremely poor [16]. In our cohort, all NECs, as included in the G3 group, demonstrated markedly limited outcomes, even in cases where aggressive therapies were applied. In addition, NECs, as well as MiNEN, showed significantly worse PFS and OS than NETs in the presented cohort. However, due to the limited cohort size, adequate subgroup analyses to identify independent prognostic factors in NEC and MiNEN were not feasible. These findings underscore the urgent need for more effective therapeutic strategies and improved early detection in this subgroup. Therefore, larger, preferably multi-center studies are needed and should be promoted.

It is widely accepted that well-differentiated rNETs smaller than 1 cm are generally at low risk of lymph node and/or distant metastases [23,24]. Multiple series have demonstrated excellent long-term outcomes for these patients, with 5-year survival rates ranging from around 95% to 100% [7,25]. Accordingly, current guidelines recommend endoscopic management when high-risk features such as G2 or G3 grading, lymphatic or microvascular invasion, or incomplete resection are absent [8]. However, exceptions to this paradigm exist: in a smaller case series, O’Neill et al. reported that 8 out of 32 patients with well-differentiated rNET smaller than 1 cm presented with lymph node involvement, and 2 even had distant metastases [9]. In our cohort, one out of the 5 patients who developed distant disease recurrence had a primary tumor smaller than 10 mm. This patient, however, had a G2 tumor, underlining that also histologic grade—not size alone—may drive aggressive behavior.

**Figure 3:**
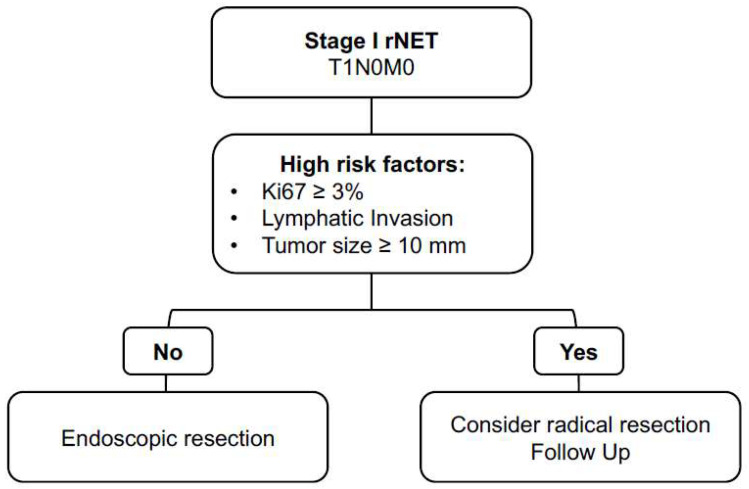
Flowchart illustrating proposed risk-stratified management of small (≤2 cm) stage I rNETs. Endoscopic resection is sufficient in the absence of high-risk factors, while the presence of one or more risk factors (Ki-67 ≥ 3%, lymphatic invasion, or tumor size ≥ 10 mm) warrants consideration of more radical resection and regular follow-up.

The optimal management strategy for rNETs measuring between 1 and 2 cm remains controversial. Tumor size has been repeatedly identified as a risk factor for metastatic behavior. Choi et al. analyzed 453 rNET patients undergoing either endoscopic or surgical tumor resection. They found that tumor size between 1 and 2 cm was associated with higher rates of lymph node metastases [26]. They additionally identified higher grade (G2) to increase the risk for nodal metastases in this group and concluded that oncologic resection should be evaluated in this group. Interestingly, while PFS was significantly shorter in these patients, no effect on OS was determined [26]. In another study, Chen et al. compared the outcome after local excision versus radical resection in rNETs between 1 and 2 cm in size and could not state a difference according to resection type [19]. They rather emphasized other risk factors associated with worse outcomes, such as age above 60 years, male sex, Black race, distant metastases, and T2 to T4 stage [19]. In the presented subgroup analysis of stage I tumors, factors that were associated with disease recurrence were lymphatic invasion, higher Ki-67 index, and tumor grade. Notably, all of these patients presented with at least one high-risk feature according to ENETS guidelines, reinforcing the importance of individualized risk stratification in the management of small rNETs [8]. Therefore, the here-proposed algorithm for stage I rNET patients is similar to the ENETS recommendations, and the evaluation of high-risk features is essential for adequate disease management, as proposed in Figure 3 below.

The development of prognostic and risk prediction models for rNETs has gained increasing attention in recent years, reflecting the need for tools that can guide individualized management. Recent studies have introduced novel scoring systems and user-friendly models that integrate multiple clinical and pathological variables to improve outcome prediction [18,27]. Recently, the GATIS score has been proposed by Zeng and colleagues. This nomogram includes the predictive factors of tumor grade, T stage, tumor size, patient age, and the prognostic nutritional index and showed higher efficacy to predict rNEN prognosis than WHO grading and tumor staging [18]. Notably, the here-presented data also identified an association of the Ki-67 index and tumor size with recurrence in small rNETs. While the strengths of the GATIS score have been mostly acknowledged, some limitations are currently discussed: by its development through a retrospective study and long-term follow-up data are missing. In addition, similar to the study presented here, molecular or genomic data have not been included [28,29,30].

There is growing evidence that beyond pathological prognostic factors, molecular and genomic markers may play an important role in outcome prediction in rNETs [31]. In this regard, expression of somatostatin receptor subtype 2A (SSTR2A) has been associated with tumor behavior and may hold prognostic as well as therapeutic relevance in rectal NETs, too [32]. In their recent study, Kim et al. were able to show that SSTR2 (Somatostatin Receptor 2) was present in 70% of rNETs and correlated with favorable clinicopathologic factors as well as improved oncologic outcomes [32]. In addition, in case of distant disease, SSTR2 expression could be used as a possible target for somatostatin analogues. Recently, investigation of genomic alterations in rNETs has revealed distinct mutation profiles between small and large rNENs [33]. Xu et al. demonstrated additionally that most investigated individuals harbored at least one genomic alteration with potential for targeted therapy, e.g., changes in the mTOR pathway [33]. Incorporating molecular and genomic markers into risk stratification algorithms could improve the precision of clinical decision-making, particularly in identifying patients who may benefit from intensified surveillance or radical treatment. Furthermore, molecular profiling may pave the road for the development of targeted therapeutic strategies, thereby complementing established histopathological criteria.

This study does have some limitations, which should be acknowledged. First, its retrospective and single-center design introduces potential selection bias and may limit generalizability. Despite pathological specimens being examined by board-certified pathologists according to a standardized protocol to determine Ki-67 indexes, some interobserver variability cannot completely be ruled out and should be considered when interpreting the results. Additionally, evaluation of the histological marker perineural invasion was mostly not available in pathology reports, preventing assessment of its potential prognostic impact. Second, the relatively small number of patients—especially in the NEC and MiNEN, and small rNET cohorts—restricted subgroup analyses and statistical power. Results from these analyses should therefore be interpreted with caution. Third, due to the low number of overall survival events, multivariable analysis for OS was not feasible. Given the rarity and heterogeneity of rNETs, it remains challenging to assemble sufficiently large and clinically homogeneous patient cohorts, even at high-volume, specialized centers. To overcome these limitations and enhance the robustness of future findings, larger multicenter collaborations and prospective registries are essential.

## 5. Conclusions

Ki-67, tumor grading, size, and metastatic status are key prognostic factors in rNENs. Even early-stage, small rNETs may carry a risk of recurrence when adverse pathological features, such as lymphatic invasion and higher Ki-67 index, are present, underscoring the need for individualized, risk-adapted follow-up strategies.

## Figures and Tables

**Table 1:** Clinical characteristics of rectal neuroendocrine neoplasms.

		Total n = 121	NET n = 84	NEC n = 26	MiNEN n = 11
Sex ratio	Female:Male	51:70	33:51	12:14	6:5
Age (years) ^1^		56 (26–81)	56 (28–81)	63 (26–79)	58 (38–69)
AJCC tumor stage	I	63 (52.1)	62 (73.8)	0 (0.0)	1 (9.1)
	II	9 (7.4)	3 (3.6)	3 (11.5)	3 (27.3)
	III	17 (14.0)	5 (6.0)	11 (42.3)	1 (9.1)
	IV	32 (26.4)	14 (16.7)	12 (46.2)	6 (54.5)
Pathological type	NET	84 (69.4)	84 (100.0)	-	-
	NEC	26 (21.5)	-	26 (100.0)	-
	MiNEN	11 (9.1)	-	-	11 (100.0)
Ki-67		3 (1–95)	2 (1–34)	80 (25–95)	70 (5–90)
Grading	1	57 (47.1)	57 (67.9)	0 (0.0)	0 (0.0)
	2	29 (24.0)	26 (31.0)	0 (0.0)	3 (27.3)
	3	35 (28.9)	1 (1.2)	26 (100.0)	8 (72.7)
Resection of primary		96 (79.3)	75 (89.3)	16 (61.5)	5 (45.5)
Complete resection (of n = 96)		62 (63.9)	44 (58.7)	14 (87.5)	4 (80.0)
Second resection (of n = 96)		24 (26.8)	20 (24.1)	2 (8.0)	2 (20.0)
Primary treatment	Endoscopic resection	66 (54.5)	63 (75.0)	2 (7.7)	1 (9.1)
	Surgical resection	31 (25.6)	12 (14.3)	15 (57.7)	4 (36.4)
	Chemotherapy	20 (16.5)	6 (7.1)	8 (30.8)	6 (54.5)
	PRRT	3 (2.5)	3 (3.6)	0 (0.0)	0 (0.0)
	Best supportive care	1 (0.8)	0 (0.0)	1 (3.8)	0 (0.0)

Values in parentheses are percentages unless indicated otherwise; ^1^ median (range); AJCC, American Joint Committee on Cancer; NET, neuroendocrine tumor; NEC, neuroendocrine carcinoma; MiNEN, mixed neuroendocrine–non-neuroendocrine neoplasm; PRRT, peptide receptor radionuclide therapy.

**Table 2:** Clinical characteristics of rNENs that underwent primary resection.

		Total n = 96	NET n = 75	NEC n = 16	MiNEN n = 5
Sex ratio	Female:Male	42:54	31:44	9:7	2:3
Age (years) ^1^		56 (26–81)	56 (28–81)	61 (33–79)	56 (46–69)
AJCC tumor stage	I	63 (65.6)	62 (82.7)	0 (0.0)	1 (20.0)
	II	9 (9.4)	3 (4.0)	3 (18.8)	3 (60.0)
	III	13 (13.5)	5 (6.7)	8 (50.0)	0 (0.0)
	IV	11 (11.5)	5 (6.7)	5 (31.3)	1 (20.0)
Pathological type	NET	75 (78.1)	75 (100.0)	-	-
	NEC	16 (16.7)	-	16 (100.0)	-
	MiNEN	5 (5.2)	-	-	5 (100.0)
Ki-67		2 (1–90)	2 (1–34)	77 (25–90)	20 (5–80)
Grading	1	55 (57.3)	55 (73.3)	0 (0.0)	0 (0.0)
	2	23 (24.0)	19 (25.3)	0 (0.0)	3 (60.0)
	3	18 (18.8)	1 (1.3)	16 (100.0)	2 (40.0)
Type of resection	endoscopic	66 (68.8)	63 (84.0)	2 (12.5)	1 (20.0)
	surgical	30 (31.3)	12 (16.0)	14 (87.5)	4 (80.0)
R status	R0	62 (64.6)	44 (58.7)	14 (87.5)	4 (80.0)
	R1	25 (26.0)	22 (29.3)	2 (12.5)	1 (20.0)
	Rx	9 (9.4)	9 (12.0)	0 (0.0)	0 (0.0)
T stage	T1	67 (69.8)	64 (85.3)	2 (12.5)	1 (20.0)
	T2	14 (14.6)	8 (10.7)	4 (25.0)	1 (20.0)
	T3	12 (12.5)	3 (4.0)	8 (50.0)	2 (40.0)
	T4	3 (3.1)	0 (0.0)	2 (12.5)	1 (20.0)
N stage	N0	10 (10.4)	4 (5.3)	3 (18.8)	3 (60.0)
	N+	21 (21.9)	8 (10.7)	11 (68.8)	2 (40.0)
	Nx	65 (67.7)	63 (84.0)	2 (12.5)	0 (0.0)
Second resection		24 (25.3)	20 (27.0)	2 (12.5)	2 (40.0)
Type of second resection	endoscopic	18 (75.0)	17 (85.0)	1 (25.0)	0 (0.0)
	surgical	6 (25.0)	3 (15.0)	3 (75.0)	2 (100.0)
Local recurrence		11 (11.5)	7 (9.3)	3 (18.8)	1 (20.0)
Distant recurrence		26 (27.1)	12 (16.0)	13 (81.3)	1 (20.0)

Values in parentheses are percentages unless indicated otherwise; ^1^ median (range); rNEN, rectal neuroendocrine neoplasms; AJCC, American Joint Committee on Cancer; NET, neuroendocrine tumor; NEC, neuroendocrine carcinoma; MiNEN, mixed neuroendocrine–non-neuroendocrine neoplasm.

**Table 3:** Clinicopathological characteristics affecting PFS in rNEN patients.

		Univariate		Multivariable	
		HR (95% CI)	*p*-Value	HR (95% CI)	*p*-Value
Female sex		1.456 (0.545–3.888)	0.453		
Age		1.008 (0.970–1.048)	0.675		
Ki-67		1.086 (1.042–1.132)	<0.001	1.071 (1.017–1.128)	0.009
Grading	G1	reference	-		
	G2	3.127 (1.195–8.184)	0.020		
	G3	6.111 (0.744–50.199)	0.092		
Tumor size		1.063 (1.007–1.121)	0.026		
T stage	T1	reference	-		
	T2	1.752 (0.494–6.220)	0.385		
	T3	5.873 (1.587–21.733)	0.008		
	T4	-			
Distant metastasis		9.668 (3.337–28.013)	<0.001	5.032 (1.615–15.683)	0.005
Positive resection margin		0.811 (0.289–2.277)	0.691		
Secondary resection		0.950 (0.311–2.903)	0.929		

PFS, progression-free survival; rNEN, rectal neuroendocrine neoplasms; HR, hazard ratio; 95% CI, 95% confidence interval.

**Table 4:** Clinicopathological characteristics affecting OS in rNEN patients.

		Univariate	
		HR (95% CI)	*p*-Value
Female sex		45.161 (0.008–247,151.159)	0.386
Age		0.997 (0.912–1.090)	0.948
Ki-67		1.108 (1.023–1.201)	0.012
Grading	G1	reference	-
	G2	7.607 (0.776–74.546)	0.081
	G3	-	
Tumor size		1.074 (0.960–1. 201)	0.214
T stage	T1	reference	-
	T2	3.264 (0.294–36.186)	0.335
	T3	-	
	T4	-	
Distant metastasis		1.000 (0.12–85.220)	1.000
Positive resection margin		2.472 (0.342–17.898)	0.370
Secondary resection		0.032 (0.000–596.541)	0.494

OS, overall survival; rNEN, rectal neuroendocrine neoplasms; HR, hazard ratio; 95% CI, 95% confidence interval.

**Table 5:** Clinical characteristics of stage I rectal neuroendocrine tumors.

		Total n = 62	No Recurrence n = 52	Recurrence n = 10	*p*-Value
Sex ratio	Female:Male	26:36	23:39	3:7	0.499 *
Age (years) ^1^		55 (28–81)	54 (28–81)	56 (33–68)	0.803
Ki-67 (%) ^1^		2 (1–16)	2 (1–6)	2 (1–16)	0.054
Grading	1	51 (82.3)	45 (86.5)	6 (60.0)	0.067 *
	2	11 (17.7)	7 (13.5)	4 (40.0)	
Type of resection	endoscopic	57 (91.9)	48 (92.3)	9 (90.0)	1.0 *
	surgical	5 (8.1)	4 (7.7)	1 (10.0)	
R status	R0	34 (54.8)	28 (53.8)	6 (60.0)	0.403 *
	R1	20 (32.3)	16 (30.8)	4 (40.0)	
	Rx	8 (12.9)	8 (15.4)	0 (0.0)	
T stage	T1	67 (69.8)	51 (98.1)	9 (90.0)	0.292 *
	T2	14 (14.6)	1 (1.2)	1 (10.0)	
Tumor size (mm) ^1^		6 (1–21)	6 (1–15)	9 (3–21)	0.280
N stage	N0	5 (8.1)	3 (5.8)	1 (10.0)	0.515 *
	N+	0 (0.0)	0 (0.0)	0 (0.0)	
	Nx	59 (95.2)	49 (94.2)	9 (90.0)	
L stage	L0	50 (80.6)	45 (86.5)	6 (50.0)	0.008 *
	L+	4 (6.5)	1 (1.9)	3 (30.0)	
	Lx	8 (12.9)	6 (11.5)	2 (20.0)	
V stage	V0	52 (83.9)	44 (85.6	8 (80.0)	1.0 *
	V+	2 (3.2)	2 (3.8)	0 (0.0)	
	Vx	8 (12.9)	6 (11.5)	2 (20.0)	
Second resection		18 (29.5)	14 (27.5)	4 (40.0)	0.457 *
Type of second resection	endoscopic	17 (27.4)	13 (92.9)	4 (100.0)	1.0 *
	surgical	1 (1.6)	1 (7.1)	0 (0.0)	
Local recurrence		7 (11.3)	-	7 (70.0)	<0.001 *
Distant recurrence		5 (8.1)	-	5 (50.0)	<0.001 *
NET-related death		1 (1.6)	0 (0.0)	1 (10.0)	0.161 *

Values in parentheses are percentages unless indicated otherwise; ^1^ median (range); * subgroups with expected count < 5; NET, neuroendocrine tumor;

## Data Availability

The datasets generated during and analyzed during the current study are not publicly available due to reasons of sensitivity and are only available from the corresponding author on reasonable request.

## Supplementary Materials

The following supporting information can be downloaded at: https://www.mdpi.com/article/10.3390/cancers17172841/s1, Table S1: Pooled logistic regression predicting recurrence depending on lymphatic (L0 vs. L+) and microvascular invasion (V0 vs. V+) after multiple imputation with 5 datasets; Table S2: Sensitivity Analysis Comparing Complete Case and Multiple Imputation Results.

## Abbreviations

The following abbreviations are used in this manuscript:

NEN	Neuroendocrine neoplasm
rNEN	Rectal neuroendocrine neoplasm
NET	Neuroendocrine tumor
rNET	Rectal neuroendocrine tumor
NEC	Neuroendocrine carcinoma
rNEC	Rectal neuroendocrine carcinoma
MiNEN	Mixed neuroendocrine–non-neuroendocrine neoplasm
PFS	Progression-free survival
OS	Overall survival
AJCC	American Joint Committee on Cancer
PRRT	Peptide receptor radionuclide therapy
HR	Hazard ration
