# Supplementary material for: Prognostic Factors in Neuroendocrine Neoplasms of the Rectum
Source: Cancers (Basel). 2025 Aug 29;17(17):2841. doi: 10.3390/cancers17172841 (PMC12427488; doi:10.3390/cancers17172841)
Supplement: Supplementary file 1 [file cancers-17-02841-s001.zip › cancers-3797602-supplementary.pdf]

Multiple Imputation Analysis

N=8 (12.9%) data missing in both variables (lymphatic invasion and microvascular invasion). Results from multiple imputation (5 imputations) were consistent with those obtained from complete-case analyses (s. Supplementary Table S1 and S2). In the logistic regression model, lymphatic invasion remained significantly associated with disease recurrence (pooled HR = 0. 046 (0.004-0.503), similar to the complete-case analysis (HR = 0.037 (0.003-0.427)), while microvascular invasion had no association with disease recurrence in the complete case and multiple imputation analysis.

Table S1. Pooled logistic regression predicting recurrence depending on lymphatic (L0 vs. L+) and microvascular invasion (V0 vs. V+) after multiple imputation with 5 datasets.

Predictor	B (SE)	HR (95% CI)	p value
L0 vs. L+	-3.085 (1.223)	0.046 (0.004-0.503)	0.012
V0 vs. V+	19.61 (24,885.036)	328386620 (0.000-.)	0.999

Note. Results were pooled across 5 imputations using Rubin's rules. HR = odds ratio; CI = confidence interval.

Table S2. Sensitivity Analysis Comparing Complete Case and Multiple Imputation Results.

Predictor	Complete Case HR (95% CI)	p value	MI Pooled HR (95% CI)	p value
L0 vs. L+	0.037 (0.003-0.427)	0.007	0.046 (0.004-0.503)	0.012
V0 vs. V+	293722798 (0.000-.)	0.999	328386620 (0.000-.)	0.999

HR = odds ratio; CI = confidence interval.
